# Investigating the efficacy of transcranial direct current stimulation on chronic pain management in endometriosis patients: A randomized controlled trial protocol

**DOI:** 10.1371/journal.pone.0306405

**Published:** 2024-08-01

**Authors:** Tatiana Camila de Lima Alves da Silva, Hégila da Silva Dantas, Luiza Eduarda Macedo, Talita Duarte Martins, Edson Silva-Filho, Rodrigo Pegado, Linda McLean, Maria Thereza Albuquerque Barbosa Cabral Micussi

**Affiliations:** 1 Physical Therapy Graduate Student, Physical Therapy Department, Federal University of Rio Grande do Norte, Natal, RN, Brazil; 2 Physical Therapy Undergraduate Student, Physical Therapy Department, Federal University of Rio Grande do Norte, Natal, RN, Brazil; 3 Ph.D, Physical Therapy, Physical Therapy Department Federal University of Rio Grande do Norte, Natal, RN, Brazil; 4 School of Rehabilitation Sciences, University of Ottawa, Ottawa, Canada; Federal University of Paraiba, BRAZIL

## Abstract

**Introduction:**

Similar to chronic pain conditions, individuals with endometriosis can be affected by central sensitization syndrome (CSS), which is characterized by a loss of analgesia and central amplification of pain. Transcranial direct current stimulation (tDCS) has shown potential as an effective intervention to improve pain generated by other chronic pain conditions impacted by CSS, such as fibromyalgia and chronic pelvic issues. This study aims to evaluate the effectiveness of tDCS on pain, fatigue, and quality of life among patients affected by endometriosis.

**Methods:**

This is a single-center, parallel, double-blinded, randomized, controlled clinical trial protocol study. We aim to recruit 40 participants affected by endometriosis (active group, n = 20; sham group, n = 20). Anodal tDCS will be delivered at an intensity of 2mA, applied over the primary motor cortex for 20 minutes per day for 10 consecutive days. There will be four assessment times: 1 week before beginning the intervention; on the 10^th^ day following the last tDCS session; and 1 and 2 months after the last tDCS session. Pain evaluated by the algometry will be the primary outcome. Pain intensity, quality of life, fatigue, and global perception of change will be the secondary outcomes. We will calculate the effects of the active versus sham stimulation on primary and secondary outcomes by using generalized estimated equations or mixed model analysis. The effect size calculation will represent the effect measure. We expect that only the active group show reductions in pain, fatigue, and quality of life. The results of this trial will produce an important first step in providing evidence on the effectiveness of neuromodulation for the management of pain and will provide data to support new studies on tDCS.

**Registration:**

Brazilian Clinical Trials Registry (RBR-4q69573).

## Introduction

Endometriosis is a recurring condition affecting 6%–10% of women during childbearing age [1]. Approximately 35%–50% of women affected by endometriosis regularly experience painful symptoms in the pelvic region [1]. The chronic, benign, inflammatory, and estrogen-dependent lesions inside or outside the peritoneal cavity characterize the endometriosis [2]. The main symptoms presented by the women are related to chronic pelvic pain, dyspareunia, dysuria, dyschezia, and irregular uterine bleeding [3]. In this sense, endometriosis has been considered a debilitating condition due to its significant impact on physical, sexual, reproductive function, sleep quality, fatigue, and quality of life [3, 4].

There is evidence showing that the chronic pelvic pain experienced by women may generate changes in pain processing involving both neural input and output [5]. The altered central adaptation or central sensitization syndrome is associated with heightened pain sensitivity, including hyperalgesia, allodynia, expanded receptive fields, prolonged electrophysiological discharge, and persistent unpleasant sensations after painful stimuli [6–8]. It is important to highlight that central sensitization syndrome does not necessarily lead to hypersensitivity to pain [9].

Therapies that directly modulate brain activity in specific neural networks may effectively alleviate chronic pain associated with central sensitization syndrome [5]. Among various central neurostimulation methods, transcranial direct current stimulation (tDCS) has recently emerged as a non-invasive, painless, and safe modality [5]. tDCS has the capability to alter cell membrane potential and cortical excitability [10], influencing the resting potential of neuronal membranes and facilitating depolarization and hyperpolarization [11]. Consequently, the current flow appears to enhance excitability in motor areas while suppressing deeper brain regions, such as the cingulate, amygdala, and accumbens, which are associated with pain perception and control [10].

Clinical trials have demonstrated the efficacy of tDCS as an intervention for chronic pain, including cases of refractory pelvic pain and primary dysmenorrhea. The authors described significant improvements in pain perception and emotional aspects of pain [5, 12–14]. These studies consistently reported a low incidence of adverse effects associated with tDCS application, describing side effects as mild and transient, including itching, tingling, headache, burning sensation, and discomfort [12–15]. However, a scoping review [16] suggested that further investigations are warranted, emphasizing the need for studies with larger sample sizes.

Despite the positive impact of tDCS on several chronic pain conditions, function, and mood [15–17] to date, only one trial reported the use of tDCS in endometriosis [18]. The authors observed an increase in the pressure pain threshold sustained for one week after the end of the treatment [18]. However, as endometriosis is a complex condition that impacts not only pain but also fatigue and quality of life, higher-level evidence mirroring clinical practice focused on women’s health to evaluate the effectiveness of the technique is needed. Also, the use of tDCS on specific brain regions may lead us to a deeper understanding of central nervous system dysfunction and recovery. Therefore, this innovative protocol holds the potential to revolutionize the endometriosis treatment involving an intervention in the central nervous system.

Considering these assumptions, this protocol randomized-controlled trial aims to investigate the effectiveness of a standardized 10-session tDCS compared to sham tDCS for pain in patients affected by endometriosis. The secondary objectives will determine the impact of tDCS on fatigue and quality of life in patients affected by endometriosis.

## Methods

### Study design and setting

This single-center, parallel, randomized, double-blinded protocol clinical trial will follow the Guidelines for Standard Protocol Items: Recommendations for Interventional Trials. This study was registered in the Brazilian Clinical Trials Registry prior to initiation (ID: RBR-4q69573). The Woman Health Lab of the Federal University of Rio Grande do Norte will store the data and after five years the principal investigator will discharge them.

### Eligibility criteria

One researcher (TC) will recruit individuals according to the following criteria: 1) age 18–40 years [19, 20]; 2) diagnosis of deep infiltration of endometriosis (confirmed by magnetic resonance imaging and/ or surgery); 3) absence of other chronic urinary tract and/or gynecological diseases/pelvic surgery, such as interstitial cystitis, polycystic ovary syndrome, hysterectomy or oophorectomy; 4) not being in the puerperal period (within 12 months after delivery, including no breastfeeding); 5) use of dienogest medication (at least 6 months of Dienogest use); 6) pain score >4, measured through the visual analogue scale; 7) cognitive and literacy ability to understand the study protocol (Portuguese language) and to answer questionnaires. We will exclude participants if they present history of brain surgery, tumors, dizziness, or epileptic seizures, own any metal implants in the head, exhibit history of alcohol or drug abuse, experience severe headaches during or after more than two tDCS sessions associated or not with dizziness, present headache (above 4 according to visual analogue scale) or severe migraine, including nausea, vomiting, sensitivity to light and sound, and possibly aura, within 6 hours after application of tDCS.

### Interventions

The researchers (HS and TD) who will not be involved in the assessments will carry out the interventions, while another researcher also blinded to group assignment, will monitor for adverse events (LE) during and after each treatment session. HS and TD will undergo training to apply and adhere to a checklist to ensure consistency across interventions. The montage must be applied and double-checked, with current flow observed every minute to ensure no impedance increase or alteration in the stimuli or electrode positioning. Additionally, the ramp-up and ramp-down must be carefully executed uniformly for all participants. The researchers (HS and TD) will conduct the tDCS interventions in a quiet and brightly lit room. All environmental conditions during sessions will remain consistent, including room temperature and lighting. During the interventions, participants will remain awake and seated in a comfortable chair with back and arm support. They may also engage in parallel activities, such as reading, and are free to converse. The researchers (HS and TD) will perform the interventions individually, in the same room. So, the environment will be the same for all volunteers.

There will be 10 consecutive tDCS sessions, lasting 20 minutes each, according to the recommendations of the Latin and Caribbean Consensus [21]. The NKL Microestim^TM^ electric stimulator will release the 2mA current through two rubber electrodes (5cm x 7cm) previously soaked in saline solution (12 mL of 150 mMols of NaCl diluted in Milli-Q water). The electrode montage will follow the 10/20 electroencephalogram coordinates. The anode electrode will be placed over the motor cortex area (C3), and the cathodal electrode over the contralateral supraorbital area (Fp2) based on standard techniques [21, 22]. Elastic bands will keep the electrodes in the correct position [22].

**Fig 1:**
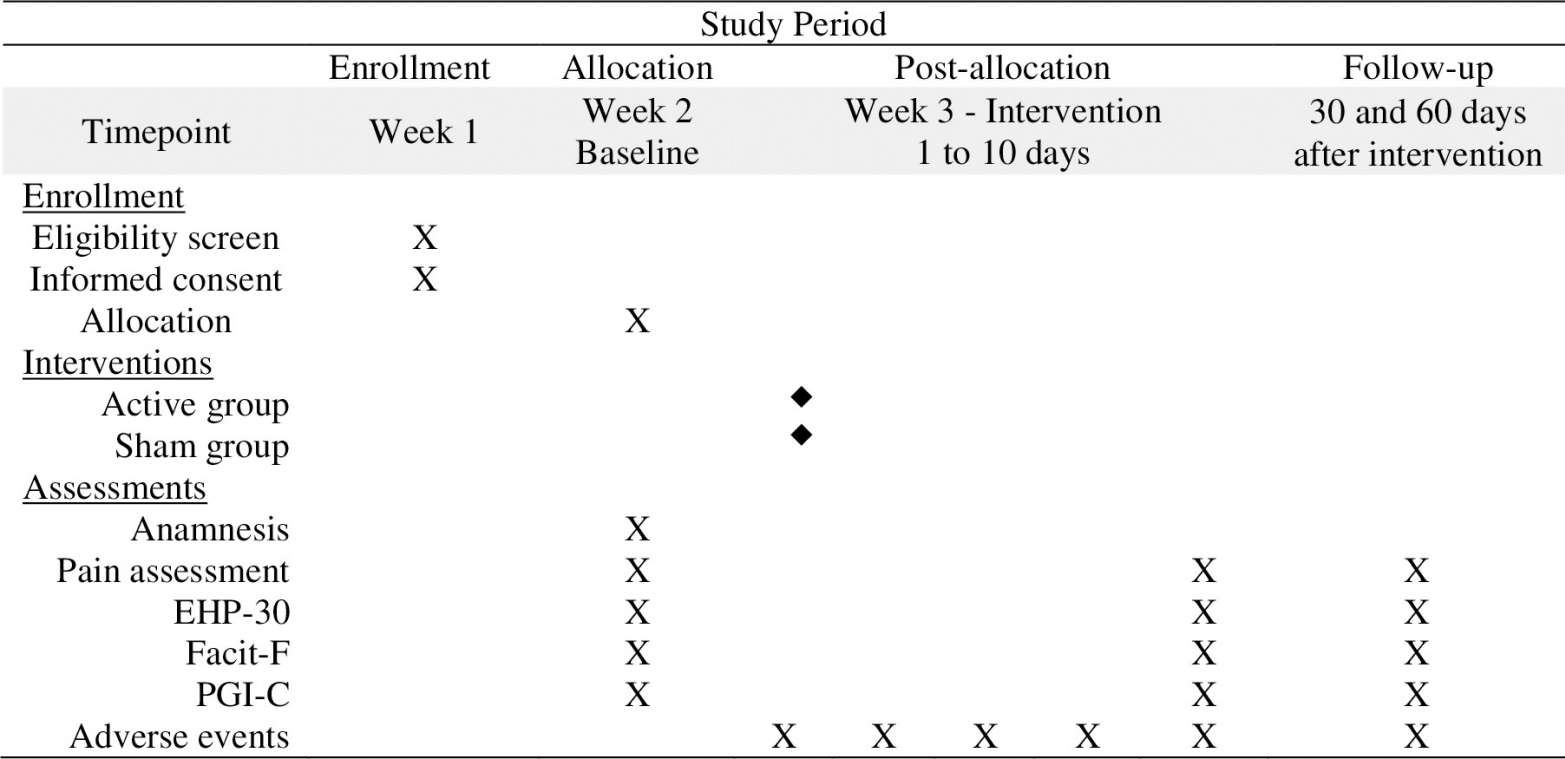
Spirit schedule. **Note:** EHP-30: Endometriosis Health Profile Questionnaire. Facit-F: Functional Assessment of Chronic Illness Therapy Fatigue Scale. PGI: Patient Global Impression.

For stimulation, 30 seconds of gradual current ramp-up and ramp-down will be performed. Subsequent tDCS sessions will be identical and will always take place at the same time of day as the initial application. The sham tDCS intervention will follow the same protocol. However, after a gradual 30-second current ramp-up to initiate the current delivery, the stimulator will be switched off during the 20-minute protocol. These methods generate the same initial sensory sensations as the active tDCS, such as itching and tingling sensations on the scalp during the first few seconds of electrostimulation [17, 23]. Fig 1 illustrates the participants’ timeline during the study (SPIRIT Schedule).

### Outcomes

**Fig 2:**
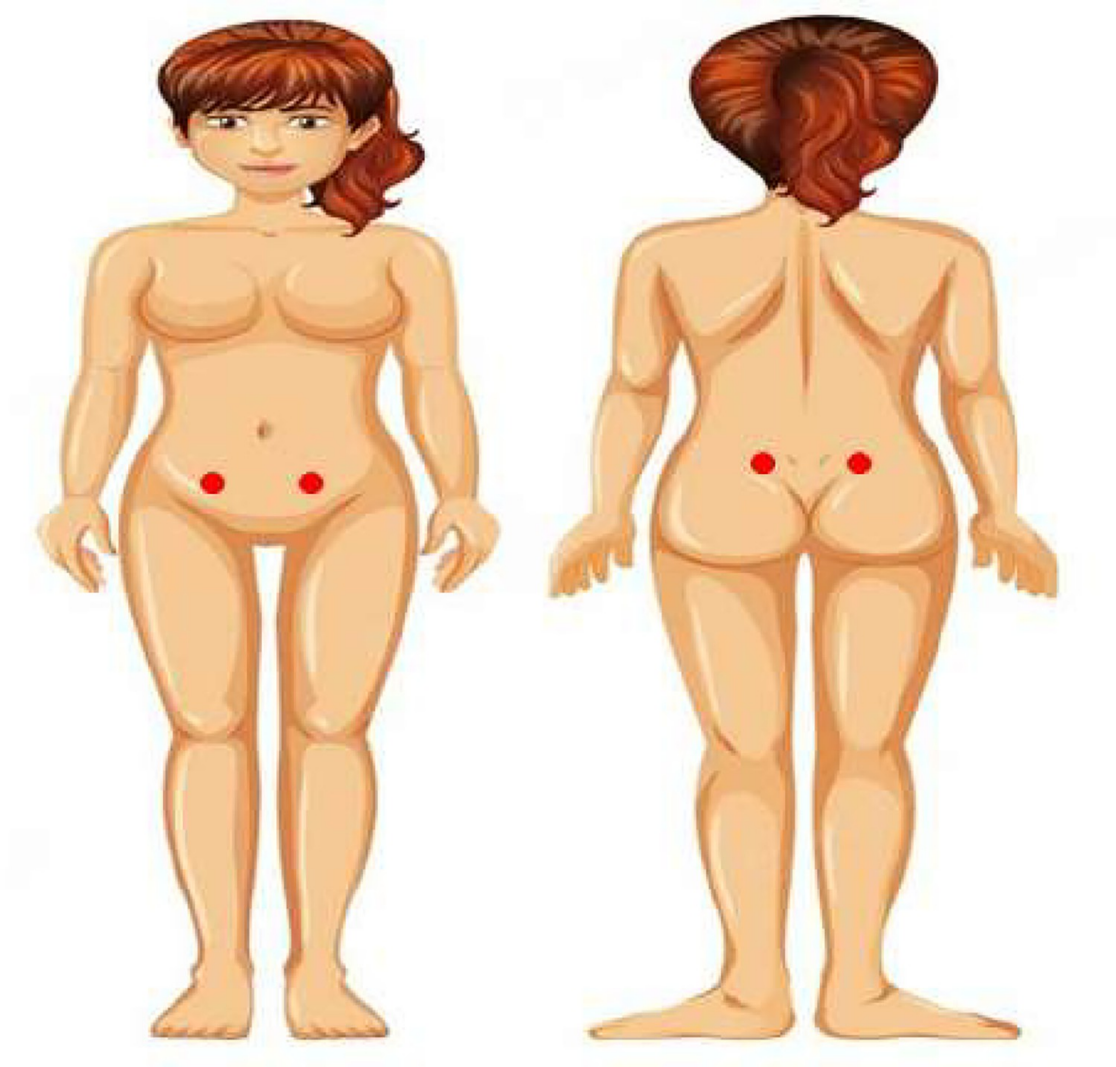
Test sites for pressure pain sensitivity testing. Red crosses indicate the local algometry in the ventral (A) and dorsal (B) regions.

The primary outcome will be patient-reported pain using the sensitivity to ventral pressure by the algometry. Pressure pain threshold and tolerance will be measured (in kg/cm^2^) by a pressure algometer (MedDor, Minas Gerais, Brazil) positioned perpendicular to the skin and applied by the same trained evaluator. The examiner will place the rubber tip (1cm^2^) over the examination area and gradually increase the pressure by approximately 1 kg/s [24]. The pain threshold will record when the patient first reports the beginning of pain perception through the phrase “It started”. For pain tolerance, the participant will sustain the maximum pressure of the algometer until reporting an inability to tolerate the pressure through the phrase “Stop” [25]. Participants must repeat exactly these two sentences for complete standardization of the exam. We will register the pain threshold and tolerance in the moment the individual starts feeling pain and does not support the pain anymore, respectively. For the perception of pain intensity, a painful mechanical stimulus will last 5 seconds through the mid-point between the pressure pain threshold and the pressure pain tolerance previously obtained from each participant. Algometry will be performed laterally on the anterosuperior and anteroposterior iliac spines (Fig 2). We will sum the results of the ventral measurement of the right and left sides to calculate the mean of the results. The calculation will be the same for dorsal [26]. It is important to note that algometry demonstrates validity and internal consistency among evaluators ranging from 0.84 to 0.99. Correlations varied from very strong (r = 0.73–0.74) to nearly perfect (r = 0.99). Inter-rater reliability yielded moderate results (r = 0.55–0.60; Cronbach’s α = 0.71–0.75) [24]. Secondary outcomes will include the assessment of pain through the visual analogue scale, Brief Pain Inventory (BPI), quality of life (30-Item Endometriosis Health Profile [EHP-30]), fatigue (Functional Assessment of Chronic Illness Therapy–Fatigue [Facit-F]), and Patient Global Impression of Change [PGI-C] induced by the intervention. Moreover, adherence (number of missed sessions), attrition, and adverse events (headache during or within 6 hours after tDCS sessions, dizziness during or within 6 hours after tDCS sessions, intolerance to electrical stimulation treatment, and any other adverse events that participants attribute to the tDCS that have not previously been reported will be recorded.

For pain assessment, we will use the visual analogue scale that presents a strong reliability for assessing chronic pelvic pain, with an intraclass correlation coefficient of 0.99 [27]. The visual analogue scale is a one-dimensional measure of pain intensity that consists of a continuous scale composed of a horizontal line, 10 centimeters (100 mm) long, anchored by two verbal descriptors, one for each extreme symptom [28]. The scale is anchored by “no pain” (score of 0) and “worst imaginable pain” (score of 100 [100 mm scale]). Participants will be asked about the worst pain on the day of assessment and will then self-complete the visual analogue scale. They will indicate on a vertical line the point that represents the intensity of their pain [28]. The visual analogue scale is the pain index frequently used by subjects with pelvic pain [29]. The BPI questionnaire is validated for the Brazilian population and assesses the intensity (domain 1) and interference of pain (domain 2) in general activities, mood, walking ability, normal work, relationships with others, sleep, and enjoyment of life. The BPI shows an intraclass correlation coefficient ICC of 0.62 for the intensity subscale, with a minimal detectable change of 2.57. For the interference subscales, the intraclass correlation coefficient is 0.76, with a minimal detectable change of 2.34 [30]. The BPI presents an 11-point scale ranging from 0 (no pain/no interference) to 10 (as bad as it can be) [31]. In addition, BPI includes a corporal diagram to assess pain location (item 2), measures the percentage of pain relief (item 8), and requests patients to describe which treatments are performed to control pain. The scores for the two dimensions range from 0 to 10 and are calculated using the mean of the total items. Higher scores represent higher pain intensity and pain interference [32].

The EHP-30 is valid, stable, and specific quality of life measure for women diagnosed with endometriosis. According to the Bland-Altman plots, the EHP-30 presents excellent test-retest reliability for measure quality of life in women with endometriosis [33]. It is a 30-item questionnaire that assesses in five dimensions: pain, control and helplessness, emotional well-being, social support, and self-image, and a modular questionnaire with 23 items distributed in six scales: sexual relationships, work, the medical profession, infertility, relationships with children, and treatment. Each dimension is evaluated in a score from 0 to 100, the result is inversely proportional to the quality of life [34].

The Facit-F scale evaluates physical fatigue, functional fatigue, emotional fatigue, and social consequences of fatigue. The Facit-F scale presents an excellent internal consistency measured by Cronbach’s coefficient α ≥ 0.88, and demonstrates an intraclass correlation coefficient ≥ 0.75 [35]. It contains 13 items with five responses ranging from “none” to “very much.” Items are scored from 0 to 4, added, multiplied by 13, and divided by the number of items actually answered. The global score ranges from 0 to 52, and higher scores reflect less fatigue [36].

The PGI-C will measure change perception. PGI-C is a simple evaluation method composed of a 7-item scale, ranging from 1 (no change) to 7 (much ​​better), in which the patient evaluates the extent to which the intervention positively impacted their condition. This scale can identify minimal clinically important changes, which are sometimes not noticed in pain questionnaires or physical examinations [37]. Madani et al. showed that the PGI-C presents a coefficient sensitivity to change of 0.85 [38].

### Participant timeline

**Fig 3:**
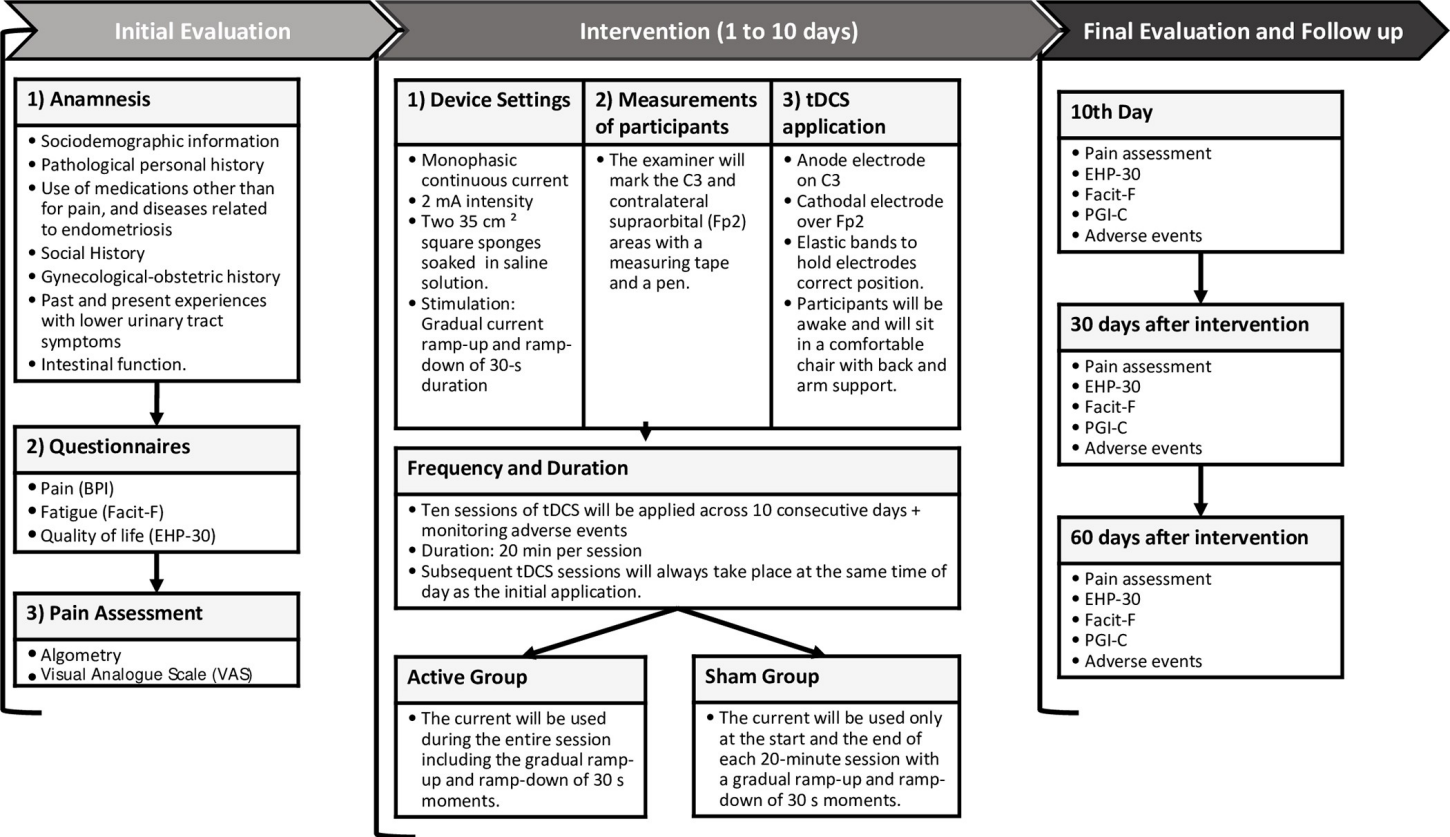
Protocol flow chart. **Note:** BPI: Brief Pain Inventory; EHP-30: Endometriosis Health Profile Questionnaire; Facit-F: Functional Assessment of Chronic Illness Therapy–Fatigue Scale; PGI-C: Patient Global Impression of Change.

Prior to randomization, all participants will undergo the initial assessment, including anamnesis, questionnaires, pain, and classification of endometriosis infiltration, according to the ENZIAN score [39]. Fig 3 details the steps of the evaluations and interventions. Two researchers (HS and TD) will administer the interventions. Another researcher (TC) will be responsible for the assessment process, ensuring consistency in the participants’ assessment. Concurrently, LE will monitor for adverse events. These procedures will be performed by trained investigators experienced in patients’ assessment affected by gynecological disorders. TC will also be responsible for the final (after 10 days of intervention) and the follow-up (30 and 60 days after the final assessment) assessments, remaining blinded to participant group assignment. These reassessments will occur concomitantly with routine consultations and the performance of imaging examinations. To confirm the participants’ attendance, researchers will call them during the week to remind them and ensure their presence. To enhance adherence to follow-up assessments, researchers will maintain an accessible support channel, available 24 hours per day via cellphone and social media, for participants to seek guidance or discuss any aspect related to their condition. Researchers will always be available to respond to participant inquiries promptly. This approach aims to ensure that participants feel supported and engaged throughout the study. Fig 1 illustrates the participants’ timeline during the study (SPIRIT Schedule).

### Sample size

The estimated sample size required for this study is based on a previous study for chronic pain [40]. The calculator is freely available at https://glimmpse.samplesizeshop.org. To determine the sample size, we employed the Hotelling Lawley Trace statistical test with a power of 0.8 and an alpha error of 0.05 for pain assessment using the visual analogue scale. We accounted for two repeated measure dimensions, with the fixed predictor divided into two groups (active and sham), considering effects for group, time, and the interaction between group and time. The expected mean values per group for pain were 6.12 and 3.06 for the active group and 4.93 and 4.37 for the sham group, with a standard deviation of 3. Assuming constant variability across time (sphericity being 1), this analysis resulted in a total sample size of 94 participants. To account for anticipated attrition, we plan to recruit an additional ten participants, totaling 104 participants (active group, n = 52; sham group, n = 52).

### Setting, recruitment strategies and data collection

This randomized controlled, double-blinded clinical trial will be conducted from December 2022 to January 2024 at the Januário Cicco Maternity Hospital of the Federal University of Rio Grande do Norte, in Natal, Brazil. Participants will be recruited from the endometriosis outpatient clinic and through social media platforms. The entire research team will utilize social media platforms to identify and engage potential participants for the study. Additionally, contact information, including a telephone number and address, will be provided for individuals interested in learning more about the study location. This will enable patients to schedule an appropriate time for their initial visit. It is important to mention that the researchers will be responsible for visiting some clinics in the city to disseminate the research and distribute posters. During the initial visit, a trained researcher (TC) will create a bond so that patients are encouraged to continue in the research and to recommend it to others. Endometriosis diagnosis will be confirmed based on examination or post-surgery reports. Cognitive and literacy abilities for study participation will be determined using the Mini-Mental test [41].

### Randomization, allocation concealment, and blinding

Participants will be randomly assigned to either the active tDCS group or the sham tDCS group in a 1:1 allocation ratio using a numerical sequence generated by freely available software (www.randomization.com). This allocation ensures that each participant has an equal likelihood of being assigned to either group. To ensure unbiased randomization, an independent researcher (RP) not involved in the assessments or interventions will conduct the randomization protocol before interventions begin. RP will then insert an identification into each opaque envelope assigned to a participant and seal the envelope to maintain allocation concealment. Only the researches (HS and TD) delivering the intervention will know the group assignment, ensuring that participants and researchers involved in assessments remain blinded to group allocation throughout the study. At the conclusion of the interventions, the blinded researcher responsible for the assessments (TC) will record the possible allocation of each participant in the assessment form. This information will enable us to calculate the percentage of correct answers and determine the effectiveness of blinding. Blinding will be considered highly effective if the correct answers amount to less than 50%. If blinding integrity is broken, we will consider it as a bias.

### Statistical analysis

Statistical analyses will be performed using SPSS software version 21.0 (IBM Corp., Armonk, NY, USA). All tabulated data will be analyzed by a blinded researcher through a sequence of randomized codes generated for data analysis. Clinical and sociodemographic characteristics will be described by means and standard deviations for continuous parameters and by frequency for dichotomous variables. By the end of the data collection, the blinded researcher and the researchers who evaluated the patients will check for double data entry and any other problem related to the data tabulation. The t-test and Qui-square test will analyze the continuous and dichotomous data for sociodemographic characteristics, respectively. The generalized estimating equations (GEE) or generalized mixed models will analyze the clinical data (primary and secondary outcomes: algometry, visual analogue scale, EHP-30, Facit-F) at baseline, final assessment (after 10 days of intervention), and follow-ups. According to the data analysis (residuals Q-Q plot, and histogram), we will choose the best distribution to represent the data. Also, we will decide to insert fixed or random factors to the constant based on A/C, B/C, and Q/C indexes, Chi-Square/DF, and the intraclass correlation coefficient. The link function will estimate the results according to the distribution of the dependent variable. Time, group (active and sham groups), and the interaction between them will represent the independent factors. The mean, mean difference, standard deviation, confidence interval, and p-value for baseline, final assessment, and follow-ups, will represent the data. It is important to mention that the PGI-C and sub-items from the BPI will be analyzed as continuous or dichotomic variables using regression analysis (linear or logistic) to explore the predictors of pain intensity and interference. The analysis will be conducted using generalized mixed models or Generalized Linear Mixed Models. We aim to verify the interaction between time and group, adjusting for confounders and modifiers effects if necessary. Also, the Wald x^2^ test will assess the significance of individual independent variables in the model. In contrast, the Bonferroni test will compare subgroups of independent variables [42]. Intention-to-treat analysis of missing data will be analyzed through follow-up data from those who do not complete the treatment protocol. If needed, we will perform imputation followed by sensitivity analysis, using the last value carried forward or group means. In the intention-to-treat analysis, we will select the most conservative value to avoid overestimating the results and confounding the analysis. For the sensitivity analysis, we will test the robustness of our results by analyzing all imputed datasets and describing any differences in the outcomes compared to the original analysis using generalized mixed models or generalized linear mixed models. If necessary, we may utilize the type III regression when dealing with unequal sample sizes and unbalanced designs that involve interactions between variables or missing data. This approach enables us to assess the unique contribution of each variable to the model’s prediction. Moreover, we will analyze and describe potential outliers based on the model’s residual plots. Additionally, sensitivity analyses may be performed to assess differences with and without the outliers. Effect sizes for interactions will be calculated for all outcomes using the partial η^2^ and mean difference. The significance level will be defined in all statistical testing as a p-value less than 0.05.

### Data monitoring

This study will not involve participants at risk of death or interventions that are harmful; therefore, it will not be necessary to create a data safety monitoring board. In case of severe adverse events that demand an interruption of the trial, the principal investigator will perform the interim analysis and will inform the ethics committee and The Brazilian Registry of Clinical Trials. The principal investigator will decide on the viability of publishing the data.

### Harms

Any adverse events will be carefully monitored in all steps of the study by asking patients, after each session of stimulation and during the follow-up periods, if they experienced any unusual symptoms or adverse events, and their perception of the relationship between these events and the treatment they received with tDCS. Any adverse event during the research period will be registered and considered in the data analysis. We will interrupt the interventions if any risk to the patients is suspected. Adverse events might include pain, fatigue, itching, tingling, headache, burning, discomfort, dizziness, and cardiovascular symptoms, such as shortness of breath, chest pain, and abnormally increased blood pressure. Although some adverse events can be observed, real-world improvements in pain, quality of life, and fatigue are expected. Although the participants are exposed to certain adverse events, the improvements particularly related to pain seem to be promising.

### Ethics and dissemination

This project was submitted to the Research Ethics Committee of the Federal University of Rio Grande do Norte and approved under protocol number 5.635.439. All participants will be informed about the research protocol and, if agree, they will sign the written informed consent before beginning any aspect of the study protocol. The study will be conducted following the principles of the Declaration of Helsinki and was registered on the virtual platform Brazilian Clinical Trials Registry (RBR-4q69573). Any personal information related to participation in the research will not be shared during or after the study. All participants will be informed about the trial’s objectives and procedures and that they will participate voluntarily, as determined by Resolution No. 466/12 of the National Health Council. After the study, all patients who received the sham tDCS will be invited to receive the active tDCS, which will be provided to them at no cost.
